# Coral persistence despite marginal conditions in the Port of Miami

**DOI:** 10.1038/s41598-023-33467-7

**Published:** 2023-04-25

**Authors:** Ian C. Enochs, Michael S. Studivan, Graham Kolodziej, Colin Foord, Isabelle Basden, Albert Boyd, Nathan Formel, Amanda Kirkland, Ewelina Rubin, Mike Jankulak, Ian Smith, Christopher R. Kelble, Derek P. Manzello

**Affiliations:** 1 Ocean Chemistry and Ecosystems Division, Atlantic Oceanographic and Meteorological Laboratory, U.S. National Oceanic and Atmospheric Administration, Miami, FL 33149 USA; 2 Cooperative Institute for Marine and Atmospheric Studies, University of Miami, Miami, FL 33149 USA; 3 Coral Morphologic, Miami, FL 33136 USA; 4 Biology Department, Woods Hole Oceanographic Institution, Woods Hole, MA 02543 USA; 5 Biological Sciences Department, University of New Orleans, New Orleans, LA 70148 USA; 6 Soil and Water Sciences Department, Genetics Institute, University of Florida, Gainesville, FL 32611 USA; 7 Satellite Oceanography and Climatology Division, Center for Satellite Applications and Research, U.S. National Oceanic and Atmospheric Administration, College Park, MD USA

**Keywords:** Biodiversity, Climate-change ecology, Urban ecology, Marine biology, Marine chemistry

## Abstract

Coral cover has declined worldwide due to anthropogenic stressors that manifest on both global and local scales. Coral communities that exist in extreme conditions can provide information on how these stressors influence ecosystem structure, with implications for their persistence under future conditions. The Port of Miami is located within an urbanized environment, with active coastal development, as well as commercial shipping and recreational boating activity. Monitoring of sites throughout the Port since 2018 has revealed periodic extremes in temperature, seawater pH, and salinity, far in excess of what have been measured in most coral reef environments. Despite conditions that would kill many reef species, we have documented diverse coral communities growing on artificial substrates at these sites—reflecting remarkable tolerance to environmental stressors. Furthermore, many of the more prevalent species within these communities are now conspicuously absent or in low abundance on nearby reefs, owing to their susceptibility and exposure to stony coral tissue loss disease. Natural reef frameworks, however, are largely absent at the urban sites and while diverse fish communities are documented, it is unlikely that these communities provide the same goods and services as natural reef habitats. Regardless, the existence of these communities indicates unlikely persistence and highlights the potential for coexistence of threatened species in anthropogenic environments, provided that suitable stewardship strategies are in place.

## Introduction

Populations of reef-building corals have been declining world-wide due to a combination of global and local stressors^1^. This macabre phenomenon is especially apparent in South Florida and the Florida Keys, where urban populations and active coastal development are in close proximity to coral reefs, influencing the goods and services they provide. Warming, acidification, poor water quality, disease, and unsustainable exploitation have contributed to a precipitous > 80% drop in coral cover throughout the Caribbean since the 1970s^2^. Currently in Southeast Florida, coral cover is less than 1%, while reefs in the Keys currently exhibit roughly 6%^3^.

The influences of the numerous stressors within the region are both temporally and spatially complex, dependent on benthic composition, hydrography, and proximity to urban development and runoff. This can lead to local refugia where, for example, seagrass photosynthesis ameliorates acidification^4^. The antithesis, however, is also true and hotspots exist whereby coral communities are exposed to extreme levels of stressors^5^. The latter situation often leads to ecosystem degradation but, in unique circumstances, extreme environments can prove valuable for science and management.

Characterization of areas with acute acidification, high temperatures, and elevated nutrients have led to insights on how these stressors may impact reefs on scales ranging from the organismal^6^ to the ecosystem^7^. In these areas, unique combinations of environmental factors can lead to outcomes that are difficult to predict using single-taxon, single-stressor studies. Particularly persistent coral species or genotypes may reveal mechanisms of resilience in the genetics of the coral host and/or the associated algal symbionts of the family Symbiodiniaceae^8^, as well as the wider microbiome^9^. These natural associations and molecular mechanisms may ultimately prove useful for increasing the efficacy and efficiency of restoration efforts and are likely critical for maintaining ecosystem function in a rapidly changing world.

While naturally-extreme coral environments have been identified worldwide in areas ranging from volcanic CO_2_ seeps^10^ and upwelling regions^11^, to mangrove channels^12^ and tide pools^13^, recent attention has focused on corals living in close proximity to metropolitan areas^14–17^. These so-called urban coral communities, which can develop on anthropogenic substrates and structures, are by their very nature relevant to reef coexistence in the Anthropocene. They are well-documented to have poor water quality, including higher turbidity, sedimentation and nutrients compared with less-impacted offshore reef systems^18–20^. It is also likely that numerous, less-studied stressors such as light and noise pollution, abrasion from litter and debris, as well as increased resource use and fishing also impact organisms in these environments^14^.

The Port of Miami is an urbanized waterway located in Southeast Florida, connecting Biscayne Bay to the western Atlantic. It is adjacent to downtown Miami and Miami Beach, and its waterways are highly engineered, incorporating seawalls, riprap, dredged canals, bridges, pilings, and piers. In addition to recreational boat traffic, it supports an active cruise industry with a total of 1220 cruise ships docked in 2019, accounting for 5,592,000 passengers^21^. In the same year, 1000 cargo ships docked in the port, moving more than a million containers and 9.6 million tons of goods valued at 27 billion USD^21^.

Expansion of the port to accommodate larger Neopanamax ships was conducted from 2012 to 2015, and included large-scale dredging extending out through the offshore reef tract that contributed to turbidity plumes reaching an extent of 228 km^2^^22^. Sedimentation associated with these efforts led to the burial and mortality of nearby corals, likely resulting in the loss of over 500,000 individuals^23^. The now Caribbean-wide stony coral tissue loss disease (SCTLD) was observed to originate in the area in 2014, causing rapid mortality of colonies^24^. Coral bleaching, owing to region-wide heat stress, also occurred in 2014 and 2015^8,25^. These more-acute disturbances have occurred on top of chronic water quality issues including eutrophication, high phytoplankton biomass, low dissolved oxygen, and acidification^5,26^.

**Figure 1:**
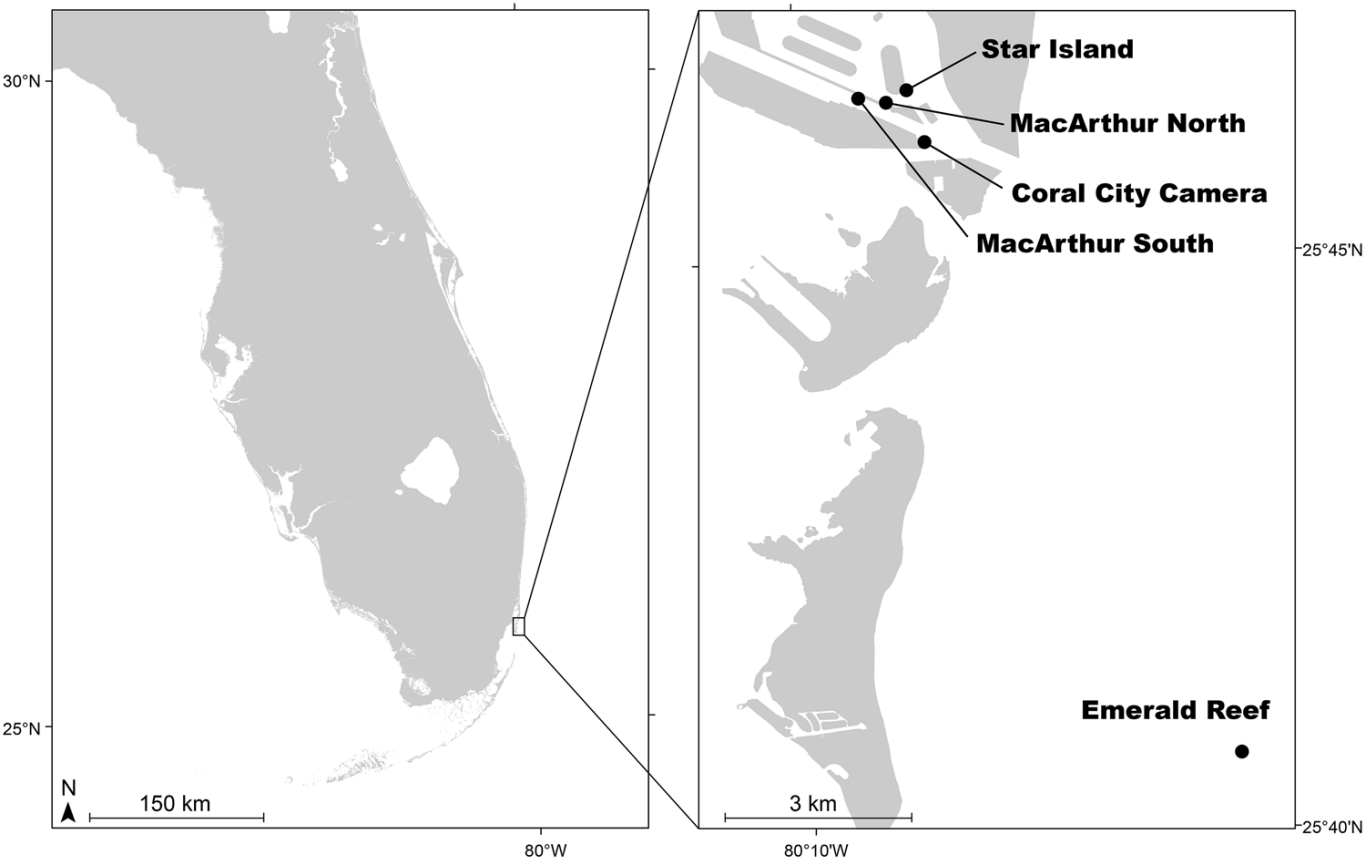
Map showing South Florida and the Port of Miami study region including the location of three inshore, urban monitoring sites (Star Island, MacArthur North, MacArthur South), the Coral City Camera site used for fish monitoring, and the more-natural Emerald Reef site. Figure produced using Illustrator (v24.3, Adobe).

Despite all these chronicled and presumed deleterious factors, corals persist. Here we document diverse coral communities living on artificial structures in the Port of Miami (Fig. 1). We describe the temporal dynamics and control of important environmental and water-quality parameters, relative to a more-natural offshore reef. These communities demonstrate remarkable resilience and represent an important source of information for understanding, predicting, and managing coral communities in a world increasingly affected by human influence.

## Results

### Environmental conditions

**Figure 2:**
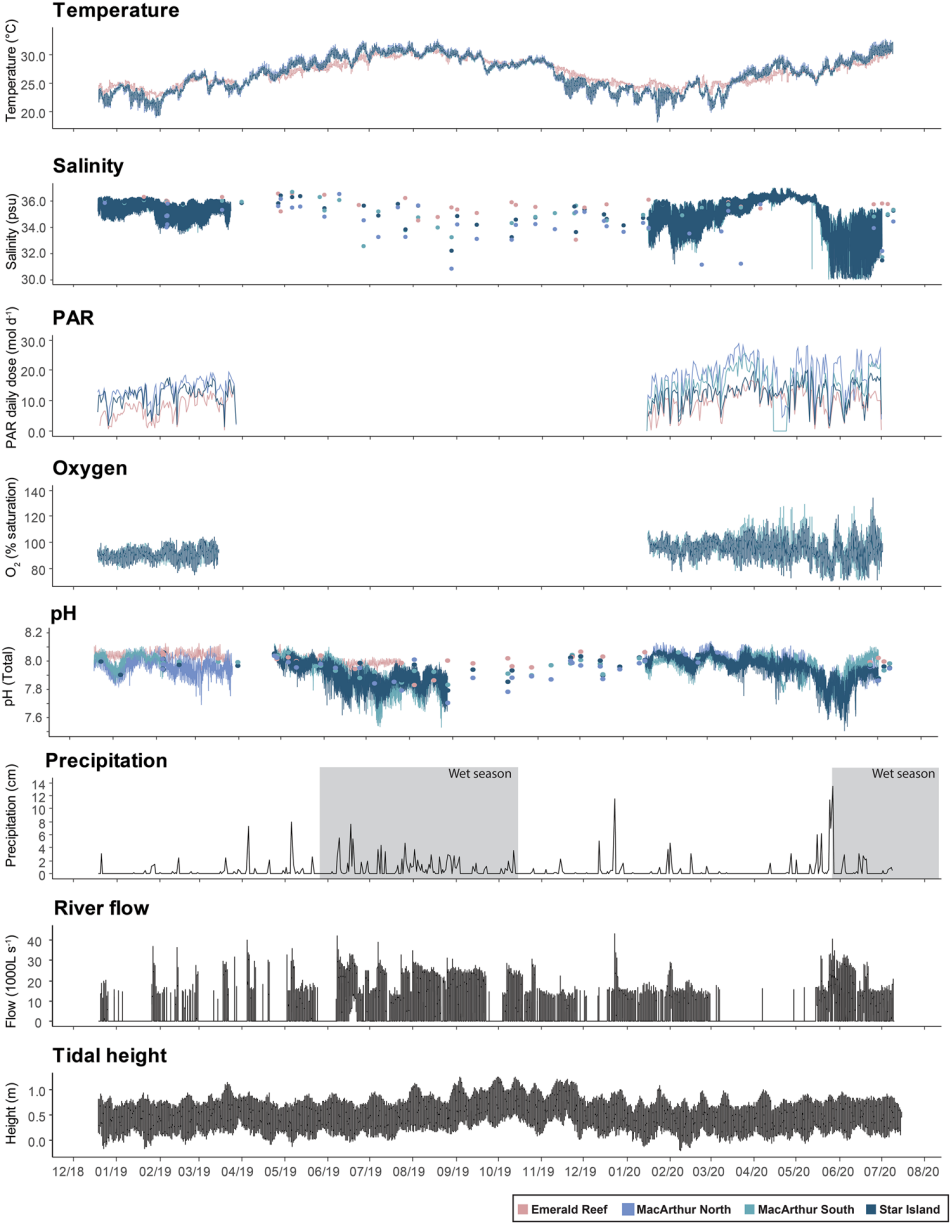
Environmental conditions at a natural coral reef (pink) and three inshore urban sites (blue) over the study’s duration. Continuous data are shown as lines, while discrete samples are shown as points (see salinity and pH data). Precipitation (cm), river flow (1000 L s^−1^) and tidal height (m) are not site-specific and are shown in black. Zeros in river flow  may reflect an absence of data, rather than no flow. PAR is photosynthetically active radiation expressed as a daily dose (mol d^−1^). Wet seasons are overlaid on precipitation data in gray.

**Figure 3:**
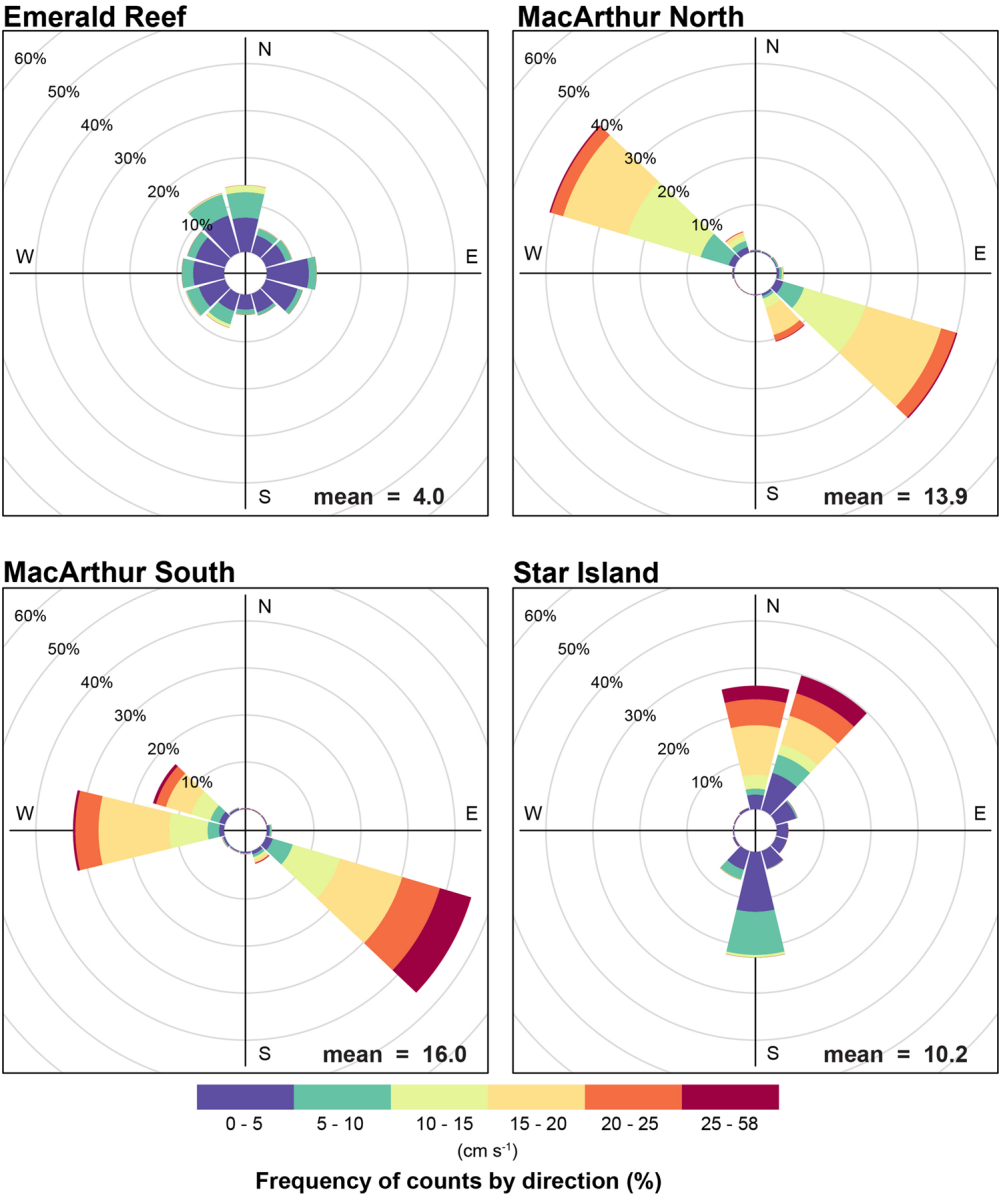
Rose plots showing the frequency and direction of current vectors by site. Mean current velocity is overlaid in the lower right of each plot.

While mean temperatures were comparable across sites (Table 1), significant differences were detected between sites and seasons (Table S1). Higher variability was observed at urban sites (Fig. 2), with more extreme low temperatures observed in the dry season and more extreme high temperatures in the wet season. The lowest (18.54 °C) and highest (32.77 °C) temperature were recorded at MacArthur North, more than two degrees colder and one degree warmer than the extremes recorded at Emerald Reef. Significant differences in salinity, photosynthetically active radiation (PAR), and flow were also detected by site and season (Table S1). In general, inshore urban sites had lower salinity than offshore, and lower values were observed in the wet season, loosely corresponding to precipitation and river flow (Table 1, Fig. 2). Light and flow were higher in the shallower, tidally-flushed urban sites than at Emerald (Table 1, Fig. 2). Currents in these sites were largely bi-directional, with MacArthur North experiencing the most uniform and MacArthur South experiencing the highest velocities (Fig. 3). Temperature and salinity fluctuations were observed to correspond to tide rather than time at inshore sites (Figs. S1–S3). While oxygen concentrations were not measured at Emerald, mean percent saturation was in the mid-90’s at the urban sites (Table 1), with high variability observed in the summer of 2020 (Fig. 2).

**Table 1:** Environmental conditions at a natural coral reef (Emerald) and three inshore urban sites (MacArthur North, MacArthur South, and Star Island), separated by season (Wet and Dry).

	Season	Temperature (°C)		Salinity (psu)		PAR (mol d^−1^)	O_2_ (%sat)	Flow (cm s^−1^)	pH (Total)			TA (µmol kg^−1^)		DIC (µmol kg^−1^)		*p*CO_2_ (µatm)	Ω_Arag_
		Sensor	Range	Sensor	Samp	Sensor	Sensor	Sensor	Sensor	Range	Samp	Samp	Stand	Samp	Stand	Samp	Samp
Emerald	Dry	25.71 (1.46)	20.70–29.46		35.77 (0.96)	8.97 (4.04)		4.1 (2.5)			8.025 (0.032)	2401.8 (23.9)	2408.6 (23.3)	2089.5 (21.1)	2103.6 (32.3)	441 (27)	3.56 (0.18)
	Wet	29.26 (0.97)	26.69–31.76		35.63 (0.55)	9.98 (3.18)		3.9 (2.9)	7.989 (0.021)	7.895–8.049	7.979 (0.065)	2368.3 (10.2)	2375.1 (13.5)	2061.1 (32.9)	2079.5 (24.0)	512 (101)	3.54 (0.36)
MacArthur North	Dry	25.18 (2.37)	18.54–30.18		34.59 (1.14)	16.04 (5.83)		13.5 (5.1)	7.986 (0.063)	7.629–8.142	7.973 (0.056)	2432.7 (63.1)	2427.5 (57.9)	2171.7 (71.7)	2151.0 (53.5)	536 (85)	3.09 (0.33)
	Wet	29.89 (1.22)	26.36–32.77		33.96 (1.48)	18.61 (6.11)		14.3 (4.9)	7.877 (0.069)	7.619–8.046	7.886 (0.087)	2372.1 (44.2)	2359.5 (35.0)	2119.7 (92.5)	2087.5 (62.0)	672 (194)	3.09 (0.55)
MacArthur South	Dry	25.31 (2.05)	20.20–29.66	35.6 (1.0)	35.49 (0.65)	14.10 (6.56)	95.3 (6.1)	16.0 (6.9)	7.989 (0.065)	7.629–8.123	8.005 (0.048)	2408.0 (41.5)	2412.8 (37.5)	2129.2 (57.2)	2138.0 (44.8)	482 (69)	3.26 (0.23)
	Wet	29.66 (1.12)	26.31–32.40	32.2 (2.7)	34.38 (1.28)	15.47 (5.31)	93.6 (9.0)	16.1 (6.8)	7.881 (0.101)	7.530–8.104	7.923 (0.046)	2361.1 (29.2)	2351.9 (34.5)	2093.3 (44.9)	2070.7 (44.4)	588 (84)	3.21 (0.30)
Star Island	Dry	25.25 (2.17)	18.14–29.82	35.5 (0.9)	35.38 (0.76)	11.86 (3.90)	93.9 (6.3)	10.3 (8.9)	7.983 (0.066)	7.634–8.127	8.008 (0.046)	2422.3 (43.4)	2426.3 (39.6)	2142.3 (60.3)	2149.9 (52.5)	480 (68)	3.26 (0.29)
	Wet	29.72 (1.15)	26.54–32.48	32.4 (2.4)	34.42 (1.31)	12.91 (4.60)	93.2 (10.4)	8.9 (7.6)	7.859 (0.084)	7.490–8.057	7.920 (0.056)	2370.7 (54.3)	2361.3 (53.8)	2103.2 (78.3)	2079.1 (63.9)	608 (116)	3.23 (0.35)

Values are means with standard deviations in parentheses, unless denoted as ranges. Values marked as Sensor are collected from instrumentation and those marked as Samp. are from discrete samples. Data marked as Stand. are salinity-standardized. Empty cells indicate periods and locations where data was not collected. The partial pressure of CO_2_ *(p*CO_2_) and the saturation state of aragonite (Ω_Arag_) are calculated from total alkalinity (TA) and dissolved inorganic carbon (DIC). PAR is photosynthetically active radiation expressed as a daily dose.

There were significant differences in pH between sites and seasons (Fig. 2, Table 1, Table S2). Urban coral sites were generally more acidic than the natural offshore site, especially during the wet season when the pH (spectrophotometric) averaged 7.886 ± 0.087 (mean ± stdev.) at MacArthur North (Fig. 2, Table 1). Urban sites also had higher pH variability, which appeared to be influenced by both light and tidal state (Table 1, Fig. 2, Fig. S1). Total alkalinity (TA) and dissolved inorganic carbon (DIC) were correlated with salinity across all sites and seasons (Fig. S2) and no diel trends were observed (Fig. S3). A property-property plot of salinity-normalized nTA and nDIC revealed a significant linear relationship with a slope of 0.76 (Fig. S4). Extremes in these parameters were observed primarily at the inshore urban sites, with lower values in the wet season and higher in the dry (Fig. S4). While TA and nTA were significantly different between seasons (higher in dry), no difference was detected between sites (Table 1, Table S2). DIC, however, was significantly different across sites and seasons (higher in dry), while nDIC was only significantly different across seasons (Table 1, Table S2). These parameters contributed to significant differences in the calculated partial pressure of CO_2_ (*p*CO_2_) across sites and seasons, with higher values observed at inshore urban sites during the wet season, and the most extreme of which was MacArthur North (dry 536 ± 85, wet 672 ± 194 µatm; Table 1, Table S2). The saturation state of aragonite (Ω_arag_) was significantly different across sites, but not seasons, with the highest values at Emerald Reef (dry 3.56 ± 018, wet 3.54 ± 0.36) and lowest values at MacArthur North (dry 3.09 ± 0.33, wet 3.09 ± 0.55; Table 1, Table S2).

With respect to nutrients, Si and NO_2_ were significantly different across sites and seasons, with higher values typically measured at inshore urban sites (Table 2, Table S3). At these sites, however, SI and NO_2_ generally had mirrored behavior, with the wet season characterized by higher SI and lower NO_2_ (Table 2, Table S3). The concentration of NO_3_ was significantly different across sites, but not seasons, with higher values again measured at inshore urban sites (Table 2, Table S3). As with the seasonal trends of the other measured nutrients, NH_4_ was higher during the wet season; however, there was no difference among sites in NH_4_ (Table 2, Table S3). PO_4_ was similar across all sites and seasons. N:P ratios ranged from 4.8 to 139.0 and were generally lower in the dry season (Table 2). Wet season values all suggested P limitation and means were greater than 120, with the exception of MacArthur North, which was 36.4.

**Table 2:** Nutrient concentration at a natural coral reef (Emerald) and three inshore urban sites (MacArthur North, MacArthur South, and Star Island), separated by season (Wet and Dry).

	Season	SI (µM)	NO_2_ (µM)	NO_3_ (µM)	PO_4_ (µM)	n	NH_4_ (µM)	n	N:P
Emerald	Dry	2.14 (2.96)	0.06 (0.10)	0.38 (0.30)	0.30 (0.45)	9	1.01 (0.99)	7	4.8
	Wet	1.42 (0.65)	0.01 (0.01)	0.23 (0.19)	0.08 (0.06)	6	10.53 (14.54)	4	134.6
MacArthur North	Dry	4.93 (3.08)	0.11 (0.06)	0.75 (0.37)	0.09 (0.09)	9	1.37 (1.71)	7	24.8
	Wet	7.24 (3.08)	0.07 (0.04)	0.60 (0.41)	0.09 (0.11)	7	2.61 (2.11)	5	36.4
MacArthur South	Dry	2.71 (1.61)	0.14 (0.14)	0.68 (0.27)	0.23 (0.53)	9	1.42 (1.34)	7	9.7
	Wet	5.27 (2.37)	0.07 (0.05)	0.68 (0.50)	0.07 (0.07)	6	8.98 (15.15)	5	139.0
Star Island	Dry	4.01 (2.69)	0.09 (0.06)	0.56 (0.29)	0.14 (0.16)	9	1.18 (1.47)	7	13.1
	Wet	5.34 (2.49)	0.06 (0.04)	0.58 (0.42)	0.06 (0.06)	7	7.09 (10.90)	5	128.8

Values are means and standard deviations are in parentheses. Sample sizes denoted as n following the respective values.

### Benthic community

**Figure 4:**
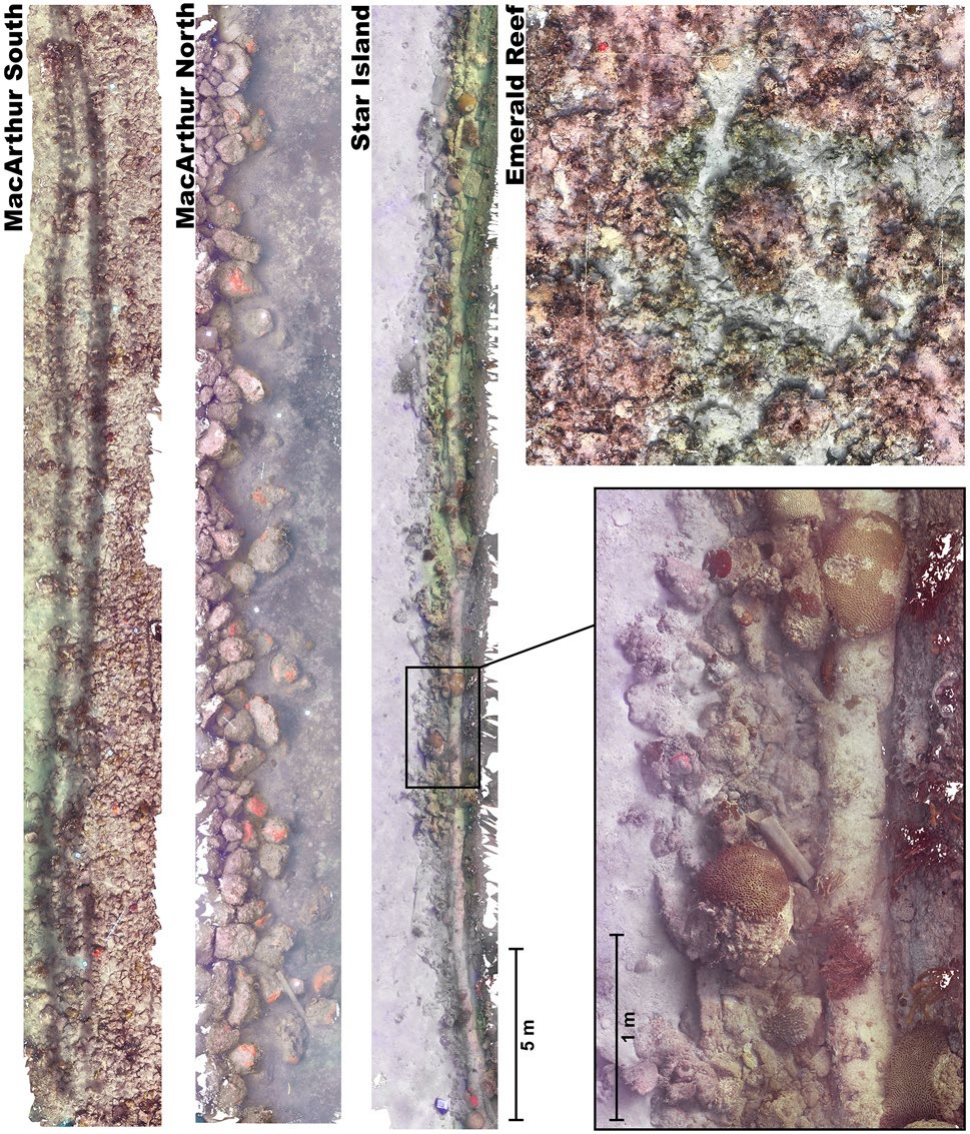
Photomosaics of each benthic study site including three elongated urban sites, as well as the natural offshore Emerald Reef. Inlay shows zoomed-in section of the Star Island mosaic. Scale bars are 5 m and 1 m in the main and zoomed-in views, respectively.

**Figure 5:**
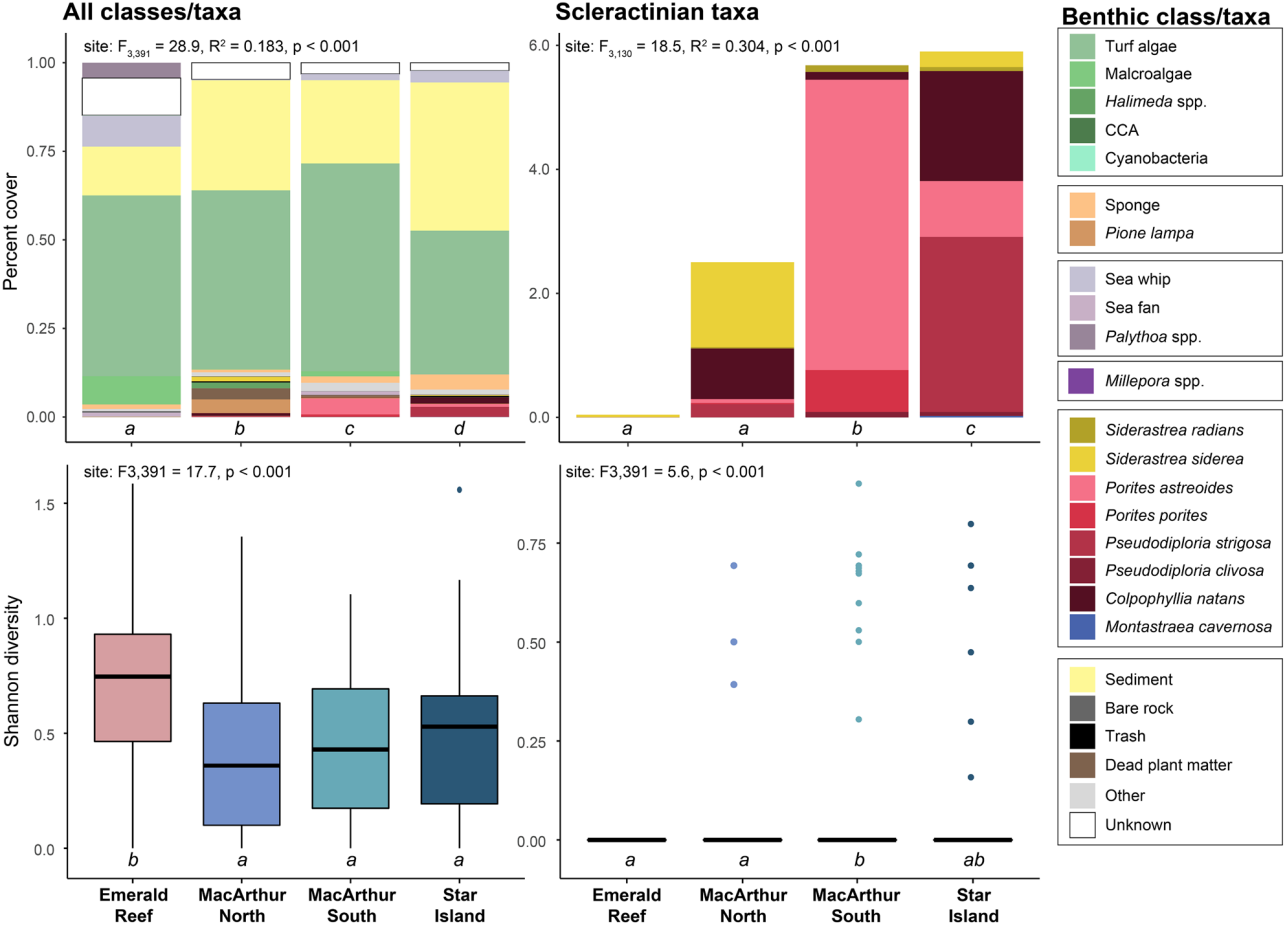
Benthic community composition and Shannon diversity metrics across offshore and urban sites (left column), with subset datasets for scleractinian taxa only (right column). Percent cover statistics represent results of PERMANOVAs, and diversity statistics represent ANOVA results. Groups which share a letter are not significantly different.

Urban sites hosted relatively diverse coral communities with significantly higher scleractinian coral cover than Emerald Reef. Urban coral communities were largely restricted to artificial hard substrates, which contributed to their linear nature (Fig. 4). Benthic communities were distinct at each of the four sites surveyed (PERMANOVA: *F*_3,391_ = 28.9, *R*^2^ = 0.183, *p* < 0.001), with all pairwise site comparisons significant (Fig. 5, Table S4). The relative cover of sediments was the most influential benthic class between all pairwise comparisons of site (percent variation ranging from 18.2 to 23.3%), with turf algae the next most influential class. When analyzing scleractinian taxa only, there was once again a significant difference in community composition among sites (*F*_3,130_ = 18.5, *R*^2^ = 0.304, *p* < 0.001), with all pairwise site comparisons significant except for Emerald Reef versus MacArthur North (Fig. 5, Table S4). SIMPER analysis indicated that few scleractinian taxa contributed to variation between sites, with *Siderastrea siderea*, *Porites astreoides*, *Colpophyllia natans*, and *Pseudodiploria strigosa* being the most differentially abundant taxa.

**Figure 6:**
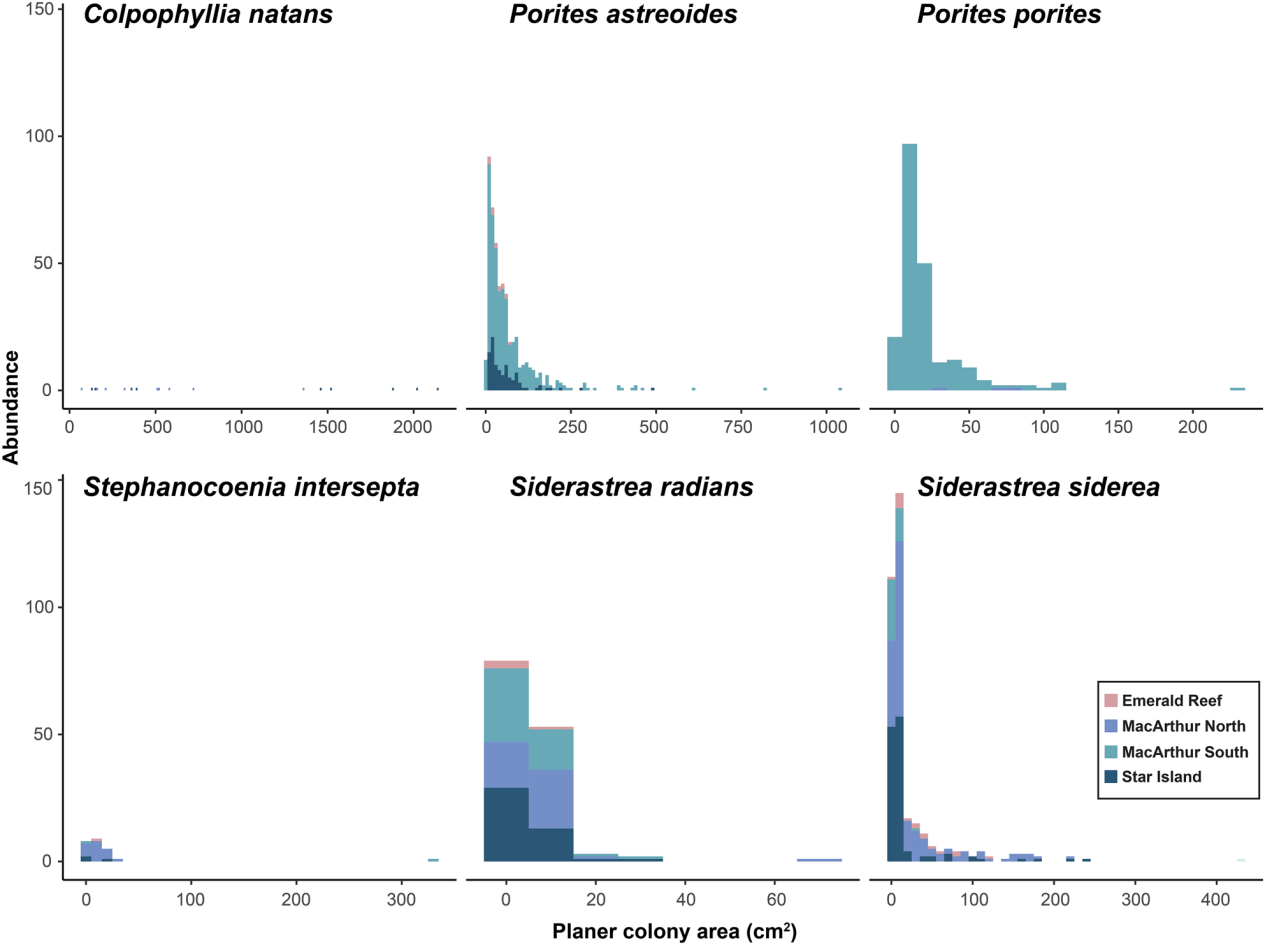
Size frequency distributions of the six most common coral species at three inshore urban sites (blue) and one natural coral reef (pink).

Overall benthic diversity was significantly higher at the natural reef site Emerald Reef compared to the three inshore urban sites (ANOVA: *F*_3,391_ = 17.8, *p* < 0.001), and analysis of scleractinian diversity only demonstrated significant variation across sites as well (*F*_3,391_ = 5.6, *p* < 0.001; Fig. 5, Table S4). Significant pairwise differences were driven by differences between MacArthur South and Emerald Reef as well as between MacArthur South and Star Island, and were likely the result of sporadic coral abundance among photos within sites. Urban coral assemblages were dominated by relatively stress-tolerant species such as *S. siderea*, *S. radians*, and *P. astreoides*; additionally, SCTLD-susceptible species, including *C. natans*, *Pseudodiploria strigosa*, and *P. clivosa*, were mainly found at the urban sites (Fig. 5). Thirteen scleractinian were observed across all urban sites, compared to eight species at Emerald Reef; additionally, coral colonies were in higher abundance at the urban sites except for *Agaricia agaricites*, which was only observed at Emerald Reef. Colony size-frequency distributions were skewed towards smaller size-classes, especially at urban sites, with the exception of *C. natans* which was sparsely distributed but reached sizes in excess of 2000 cm^2^ (Fig. 6). Massive and encrusting morphologies were dominant at the inshore sites, though abundant *Porites porites* (branching) was recorded at MacArthur South. Unlike scleractinians, gorgonians were observed to be more abundant at Emerald Reef versus inshore sites.

### Fish composition

A total of 139 species of fish were identified, belonging to 48 families (Table S5). Many of these species are known associates of coral reefs in the area. While 28 of these species were observed on a near-daily basis, 31 were observed five or fewer times including the critically-endangered smalltooth sawfish (*Pristis pectinata*).

## Discussion

The coral cover at the urban sites (2.50–5.90%) was higher than at the natural coral reef control site (0.04% ± 0.30) and higher than the mean cover of Southeast Florida coral reefs in 2020 (0.8% ± 0.21)^3^ regardless of the multitude anthropogenic stressors. Reefs in the region stopped accreting between 3700 and 8000 years ago^27^ and are, therefore, more accurately characterized as coral communities today. While *Montastraea carvernosa* were dominant in the region in the early 2000’s^28^, today *S. siderea* and *S. radians*, as well as *P. astreoides* and *P. porites*, are among the most common species, similar to the urban sites recorded herein^3^. These species, with the exception of *S. siderea,* have been previously documented within Biscayne Bay^29^. Despite significantly higher coral cover and species diversity at the urban sites relative to the natural reef site, coral diversity was still near-zero for all sites examined. This highlights the sporadic nature of coral benthic cover on the artificial substrates in the Port of Miami, and conversely emphasizes the relative low abundance of reef-building species at the reef site.

Notably absent or rare at our reef control site and at many offshore reefs in the region^3^ are massive species that are especially susceptible to SCTLD (e.g. *C. natans*, *P. strigosa*, *P. clivosa*)^30^, which is believed to have originated in the region during the time of Port of Miami dredging in 2014^24,31^. By contrast, we recorded numerous colonies of *C. natans* and *Pseudodiploria* spp. living on the artificial substrates of the port, including the largest individuals observed in this study. While their persistence relative to offshore populations is notable, massive and encrusting morphologies are known to dominate in turbid, human-impacted reefs elsewhere^14,32,33^.

Coral cover at urban sites was comparable to that of the Florida Keys (6.1% ± 0.87, 2018)^3^ and is likely too low to lead to the development of frameworks through active reef accretion^34^. While species may continue to thrive in these habitats, it is therefore likely that they will not provide the same level of anthropocentric ecosystem goods and services as natural reef systems^14,35^. We observed, however, numerous species of fishes, including many known to be reef-associates, within the port, suggesting that these urban coral communities may be providing some of the same ecological services as natural systems.

### Unfavorable environmental conditions

The occurrence and persistence of coral communities in these urban environments is especially notable given the marginal environmental conditions. While temperatures between the sites were significantly different (Table S1), means were similar across sites within seasons (~ 25 °C, Dry; ~ 29 °C, Wet). MacArthur North and Star Island, however, experienced particularly extreme cold temperatures (~ 18 °C) in the dry season, and all maximum temperatures experienced during the wet season at all urban sites exceeded those at Emerald by up to 1 °C. Extreme temperatures at these sites were correlated with low tide (Fig. S1), when shallow depths and reduced water movement would have resulted in a greater influence from local weather conditions. Higher seasonal temperature variability has been documented in nearby Biscayne Bay and is also likely influenced by reduced water exchange^29^. A similar pattern of temperature extremes in navigational inlets versus offshore reef sites has been documented in South Florida, though the trend was not significant, likely due to low sample sizes^5^. Interestingly, MacArthur South, which is located within the Port of Miami navigational inlet, experienced thermal conditions more similar to offshore sites, likely due to greater water exchange versus MacArthur North and Star Island, which are separated from the open ocean via the MacArthur Causeway.

Both warm^36^ and cold-water^37^ events are well-known to cause bleaching and coral mortality. Global warming has resulted in a significant increase in extreme warm events since the 1970’s when in situ records were initiated on coral reefs in the Florida Keys, and if trends persist, annual bleaching may occur in the region before 2034^25^. If biological and environmental resilience mechanisms are not sufficient to keep pace with this level of warming, these coral communities may succumb to warming, bleach, and eventually die.

Urban coral communities were exposed to more acidic conditions than natural reef environments in the region, with mean conditions that are not expected to occur globally until decades in the future. These data are supported by Enochs et al.^5^, who documented acidification in water emanating from navigational inlets in South Florida, including the Port of Miami. Our data also indicate a negative correlation of TA and DIC with salinity, and a nearly identical TA/DIC slope. This study extends further inshore and, unlike the prior study, used high-frequency instrumentation to document that this acidification is highly variable in nature, and in addition to diel fluctuation, is influenced by tidal state (Fig. S1). These data, therefore, support the hypothesis that the acidification may be driven by organic matter enrichment and nutrients from interconnected freshwater canals and rivers^5^.

Regardless of the particular driver(s) of this coastal acidification, low-pH conditions are known to impede calcification and accelerate bioerosion while encouraging macroalgae proliferation, thereby limiting framework development at urban sites^7,38^. The high variability measured at these sites, however, may provide a respite from acidification stress and result in physiological responses different than under sustained low pH^39,40^.

In addition to coastal acidification, the urban coral environments had higher concentrations of Si, NO_2_, and NO_3_. Poor water quality may also affect community composition through elevated nutrients, organic matter, pathogens, contaminants, turbidity, and/or sedimentation^41^. The particular agent(s) involved may be difficult or impossible to separate and identify^41^. High N:P ratios across all sites, particularly in the wet season, may indicate phosphate limitation, which has been linked to reduced photosynthetic efficiency, paling, low symbiont density, as well as reduced growth and greater density skeletons in corals^42^.

Previous work in the region has documented that five canals, Munisport Landfill, and the surrounding urban environment contribute to high turbidity, phytoplankton, and nutrients^26,43^. These waters transit through the Port of Miami, where they contribute to poorer water quality outside of the navigational inlet^5^. Sedimentation and subsequent burial of corals, along with salinity fluctuations, have been shown to limit coral community development in Biscayne Bay, and it is likely that similar processes influence the urban coral environments described herein^29^.

Similarly poor water quality conditions appear to be a common characteristic of urban coral environments, though their impact can vary^14^. Seminal studies in Hawaii have shown how improperly treated sewage effluent can lead to the degradation of nearby coral reefs^44^. Water quality parameters influenced by nearby human activity were found to explain variation in coral condition in Singapore^45^ and benthic composition in Borneo^33^. In Colombia, coral reef communities have been discovered at the mouth of Cartagena Bay, with high industrial and sewage pollution, as well as sedimentation^46^, though poor water quality has contributed to mortality and physiological impairment^47^.

### Environmental mechanisms of persistence

The proliferation of anthropogenic coastlines, termed ocean sprawl, has transformed the ecology of coastal marine ecosystems. These artificial structures are fundamentally different in both their structure and orientation, often manifesting as homogeneous vertical hardscapes versus gradually sloping shorelines with heterogeneous microhabitats and soft sediments^48^. This is especially true in South Florida where natural rocky intertidal areas are relatively uncommon. While the vertical nature can reduce the intertidal zone, it may confer benefits to sessile subtidal invertebrates such as corals. For example, benthic stability is critical for slow-growing, long-lived organisms. Disturbance of unconsolidated substrates from wave action and storms can lead to burial, mortality, and the availability of uncolonized substrate, promoting faster growing non-calcareous species. Vertical surfaces are less likely to accumulate sediments, and organisms growing on their surfaces may in turn be conferred the same benefit, resulting in less burial and a reduced metabolic cost of clearing surfaces. Finally, vertical and overlying structures may help to shade corals, reducing the potential for bleaching under elevated temperature^49^.

Increased turbidity, driving reduced water clarity, may serve the same shading function and provide indirect refugia from bleaching or reduce algae proliferation^32^. Areas such as northwest Borneo that have high turbidity due to land-based sources of pollution and development, have had lower bleaching and high recovery following thermal stress^33^. It is, however, unlikely that this is the principal driver of community persistence in the Port of Miami considering that urban habitats are shallower and have higher light (Table 1), coupled with greater temperature extremes.

### Biological mechanisms of persistence

Biological mechanisms may be contributing to coral persistence in the Port of Miami, despite temperature extremes, acidification, salinity fluctuations, turbidity and sedimentation, as well as eutrophication, and the potential for pathogens and contaminants.

Supplemental nutrition through heterotrophy contributes to colony health^50^ and has been identified as a means of ameliorating acidification^51^ and thermal stress^52^. High plankton biomass in urban environments may, therefore, contribute to the growth and persistence of corals in these settings, as observed through isotopic source tracking of autotrophic vs. heterotrophic energy sources^53^. Indeed, a prior study on the gene expression of *P. strigosa* in the Port of Miami found upregulation of genes that allow them to detect, recognize, and digest food^54^, and continued examination of trophic strategies in Miami’s urban coral populations is warranted. In addition to corals, however, high food availability may stimulate populations of suspension-feeding organisms that are less-desirable for reef development, such as the bioeroding clionaid sponges that were found to be present at urban sites herein (Fig. 5, Table 3)^11^.

**Table 3:** Percent cover of the benthos at a natural coral reef (Emerald) and three inshore urban sites (MacArthur North, MacArthur South, and Star Island).

	Abiotic	Macro-algae	Turf algae	CCA	Clionaid sponges	Hard coral	Cyano	Fire coral	Soft coral	Sponge	Other
Emerald	15.94 (19.14)	8.89 (9.27)	57.02 (19.41)	0.04 (0.31)	0.00 (0.00)	0.04 (0.30)	0.21 (0.82)	0.04 (0.30)	15.70 (18.14)	1.43 (2.73)	0.68 (3.44)
MacArthur North	33.07 (32.5)	1.83 (7.78)	52.95 (34.82)	0.00 (0.00)	4.08 (9.35)	2.50 (5.42)	0.06 (0.47)	0.00 (0.00)	0.23 (1.00)	0.80 (2.61)	4.47 (9.75)
MacArthur South	24.38 (22.35)	1.47 (3.11)	60.56 (18.18)	0.00 (0.00)	0.15 (0.99)	5.68 (8.60)	0.00 (0.00)	0.00 (0.00)	2.86 (6.85)	1.89 (4.31)	3.02 (5.27)
Star Island	43.26 (30.12)	0.04 (0.29)	41.47 (24.38)	0.00 (0.00)	0.02 (0.20)	5.90 (13.99)	0.00 (0.00)	0.00 (0.00)	3.58 (7.86)	4.35 (6.72)	1.37 (3.25)

Abiotic is inclusive of bare hard bottom, sediment, and trash. CCA is crustose coralline algae, Cyano. is cyanobacteria. Standard deviations are in parentheses.

Urban coral environments are characterized by especially high environmental variability, which may be instrumental in the resilience of reef organisms under climate change^55^. Temperature fluctuations were observed to be correlated with the tidal cycle (Fig. S1) and similarly influenced back reef environments in American Samoa have been shown to be home to more thermally-tolerant corals^13^. The benefit of this temperature variability is likely due, in part, to mechanisms of acclimatization that are not directly related to symbiont shuffling, as has since been demonstrated experimentally both in the field^56^ and laboratory^57^. For example, transcriptomic examination of Port of Miami corals found higher expression of immune-related pathways relative to natural reef corals, suggestive of immune/stress front-loading or priming^54^. Seawater carbonate chemistry in the Port of Miami was also observed to be highly variable across seasonal and diel cycles, with periodic exposure to pH conditions more extreme than those projected to occur in open ocean waters at the end of the century due to global OA (Table 1)^58^. Under contemporary conditions, these fluctuations may lead to enhanced calcification and coral growth, contributing to their persistence^39^, as has been postulated at other acidified reefs with high variability^59^.

The resilience of urban coral communities may be due in part to the composition and physiology of their symbiotic associates. For instance, Rubin et al.^54^ found that *P. strigosa* in the Port of Miami primarily hosted more heat-tolerant zooxanthellae in the genus *Durusdinium* vs. those at our natural control site, Emerald Reef, which primarily hosted *Breviolum*. Similar associations with stress-tolerant symbionts have been observed in urbanized habitats in Singapore^60,61^, as well as in environmentally-variable lagoon environments^62^. Prokaryotic communities also tend to reflect the environment in which the coral resides, where corals in marginal habitats can host divergent mucus-associated bacteria^63^. While some host-symbiont associations in urbanized environments may ultimately be beneficial to the coral host, shifts in prokaryotic communities due to urbanization (i.e. loss of microbial diversity compared to natural reef corals) may instead be detrimental^64^. Likewise, the previous study also observed disruptions to seasonal and gametogenic cycles in the coral host, which may have implications for the ability of urban corals to contribute larvae to persistence of local populations.

Beyond specialized host/symbiont interactions in urbanized environments, it is generally less well known how host genotype identity may contribute to coral resilience due to difficulties in identifying molecular bioindicators of resilience^65^. Further, genotype x environment effects are often confounded in wild populations without the use of manipulative experiments^66^. In at least one case reported in the Red Sea, however, consistent exposure to temperature extremes was hypothesized to have resulted in the selection of heat-resilient coral genotypes through time^67^. In the Port of Miami, where SCTLD was observed to decimate nearby offshore populations starting in 2014 to the present^24,68^, urban coral populations have been largely spared from disease-related mortality. Whether this observed phenomenon is due to proximity within the Port, disease-resistant urban genotypes, or algal symbiont/microbial communities remains to be tested. It is likely that there exists a hierarchy of resilience vs. susceptibility to stress within and among coral species^16^, in part due to host genotype, algal symbiont and microbial associations, and physiological and molecular mechanisms described above. This, in turn, requires a holistic understanding of coral resilience and accurate methods to evaluate genotype performance through the use of high-resolution genotyping and symbiont typing approaches, examination of physiological tolerance and disease resistance, and evaluation of phenotypic plasticity vs. adaptation through manipulative experimentation.

### Broader context and future research needs

Coral mortality and declines in reef ecosystem health have become commonplace the world over largely due to anthropogenic climate change and unsustainable development. The existence of urban coral communities in the Port of Miami, an apparently adverse environment engineered for industry, is therefore remarkable. These corals may be uniquely suited to exist in these conditions due to their genetics, or they may be capitalizing on more ubiquitous mechanisms of resilience. They may, therefore, be at the brink of peril on the edge of their environmental tolerance or provide a glimpse into persistence and coexistence. Regardless, they provide insights that could be applicable to ever-increasingly urbanized marine environments, the so-called ocean sprawl^48^.

While the unique ecology and behavior of animals living in terrestrial urban environments has been well studied, along with their management and conservation^69^, these topics are poorly understood in the marine realm. Further, city planning and urban development are often conducted with principles of conservation that provide services to wildlife and human populations alike^70^. Urban marine environments can likely be engineered with an ecological perspective that leverages coexistence towards structural persistence, as well as ecosystem services^71^. For example, where urban habitats may host sexually mature coral populations, they may constitute refugia through self-seeding and potentially the export of larvae to nearby reef environments.

In order for urban areas in the Port of Miami to serve as refugia, hydrodynamic and genetic connectivity must be established with more natural reef environments^72^. While offshore reefs have the capacity to seed urban substrates, as evidenced by coral recruitment on artificial substrates, the ability for these populations to serve as a source has yet to be established. Complex hydrodynamic patterns, such as those driven by tidal cycles within inshore and port environments, may result in low gene flow and the development of cryptic urban lineages. In Singapore, for example, urban populations were found to be genetically distinct from natural reef populations, with a majority of individuals representing nonmigrants^73^. Urban populations may conversely be largely self-seeding with little gene flow to offshore reefs, but this hypothesis requires examination with population genetics approaches. Further, it is not yet known whether coral genotypes currently present within the Port are more resilient to future environmental conditions than their offshore counterparts, and if they are, whether it is persistent due to inherent genetic advantages or alternatively fleeting due to unique environmental circumstances. Increasing connectivity could help to confer advantageous traits to populations in natural reef environments, though potentially deleterious factors such as the spread of disease should be considered.

Finally, while the resilience and prevalence of coral communities in the Port of Miami are remarkable, their long-term persistence, and therefore capacity to serve as a future source of genetic material is, at this time, questionable. By their very nature and proximity, they are more subject to urban development. Maintenance or enhancement of the same infrastructure that originally provided habitat has the potential to destroy it. This was particularly evident in June 2022 when the seawall at the Star Island site was observed to have toppled, resulting in complete mortality of the associated communities including the ESA threatened species *Orbicella faveolata* and disease-susceptible brain coral species, and partial mortality of colonies in adjacent communities due to siltation and burial. Regardless, these coral communities serve as a reminder of the potential for coexistence on an otherwise depressing tableau of global change, widespread reef degradation, and mass extinction. Going forward, the close collaboration of permitting and governing agencies, environmental stewards, and developers is critical for ensuring that the current and future environmental value of these ecosystems is responsibly balanced with the economic value of the infrastructure on which they have developed.

## Methods

Three study sites were located along engineered seawalls and riprap (~ 0.5–2 m depth) within the highly urbanized Port of Miami (MacArthur South, MacArthur North, Star Island, Fig. 1). A fourth site, indicative of a more-natural coral reef, was located roughly 12 km away at Emerald Reef (~ 7 m depth, Fig. 1). Monitoring was conducted from December 2018 to July 2020 in order to characterize the unique environmental conditions and biological communities present at each site. Observations spanned both wet and dry seasons, occurring approximately late May through early October and mid-October through mid-May, respectively, though not all datasets were continuous throughout the duration due to various interruptions.

### Environmental conditions

Temperature was measured at each site every 5 min using high-accuracy thermistors (SBE56, Seabird Scientific). Salinity was inferred from conductivity and temperature loggers (SBE 16plus V2 SeaCAT, Seabird Scientific), as well as from bottle samples analyzed with a densitometer (DMA 5000 M, Anton Paar). PAR was measured every 30 min at each site using ECO-PAR™ loggers (Seabird Scientific), which includes a sensor-cleaning mechanism to reduce measurement drift associated with fouling during long-term deployments. Instantaneous PAR readings, which are time-dependent, were summed between the hours of 10 am and 3 pm, to calculate a daily dose. Dissolved oxygen was measured during select deployments of the SBE 16Plus meters, using attached dissolved oxygen optodes (SBE 63, Seabird Scientific). Current speed and direction were recorded every 15 min using tilt current meters (Lowell Instruments).

Carbonate chemistry was characterized at each site using discrete water samples and SeaFET™ pH loggers (Seabird Scientific). Discrete water samples were collected at depth by divers directly into borosilicate glass bottles which were immediately fixed at the surface using 200 µL of HgCl_2_ and sealed using Apiezon grease and ground glass stoppers. Discrete seawater samples were also collected in between field excursions using Subsurface Automated Samplers^74^ programmed to collect water 2 h after the first high tide of the day. Samples were immediately fixed using 200 µL of HgCl_2_ and kept in CO_2_-impermeable sampling bags until retrieval (< 1 mo), when they were transferred to, and sealed in borosilicate glass bottles as above. Total-scale pH was measured spectrophotometrically. TA was measured using automated Gran titration (AS-ALK2, Apollo SciTech) and DIC was determined by stripping and measuring the CO_2_ gas in the water sample (AS-C3, Apollo SciTech). TA and DIC values were measured twice and corrected using certified reference materials following Dickson et al.^75^. Ω_Arag_, *p*CO_2_, and pH (when not measured directly) were calculated using Seacarb^76^ with the constants recommended in Dickson et al.^75^. TA and DIC were regressed against salinity and then salinity-standardized using a non-zero endmember following Friis et al.^77^.

SeaFET sensor performance was evaluated and data were only used during periods of continuous normal operation. Sensor readings were corrected using offsets calculated from the mean of discrete water samples collected at each site during the same deployment (n = 2–9). Offset calculations included water samples that were not used for the comparison of carbonate chemistry between sites.

Samples for nutrient analysis were collected by hand at the same time as discrete carbonate chemistry samples. Samples were first filtered using a nylon syringe filter (0.45 µm) and 50 mL was transferred to each of two seawater-rinsed 60 mL plastic vials. Both samples were kept on ice in the field. The vial reserved for analysis of ammonium was fixed with chloroform (~ 0.2 mL) and was transferred to a refrigerator within hours of collection. The vial reserved for analysis of other nutrients was frozen within hours of collection.

Dissolved nutrients were measured using a five-channel automated continuous flow analytical system with segmented flow and colorimetric detection (SEAL Analytical). Analysis was carried out for nitrate (NO_3_^−^), nitrite (NO_2_^−^), phosphate (PO_4_^−3^), orthosilicic acid (H_4_SiO_4_), and total ammonia (NH_3_ and NH_4_^+^). Nitrite was determined by diazotizing the sample with sulfanilamide and coupling with N-1 naphthyl ethylenediamine dihydrochloride to form an azo dye. Samples for nitrate analysis were passed through a cadmium column, which reduced nitrate to nitrite, and the resulting nitrite concentration (i.e. nitrate + nitrite, N + N) was then determined as described above. Nitrate concentrations were determined from the difference between N + N and nitrite^78^. Phosphate was determined by reacting the sample with molybdic acid to form phosphomolybdic acid. This complex was subsequently reduced with hydrazine, and the absorbance of the resulting phosphomolybdous acid was measured^79^. Silicic acid was analyzed by adding an acidic solution of ammonium molybdate to seawater to produce silicomolybdic acid^80^. Oxalic acid was added to inhibit a secondary reaction with phosphate and ascorbic acid was added to form silicomolybdous acid, which was measured colorimetrically. Ammonia in solution reacts with alkaline phenol and NaDTT at 37 °C to form indophenol blue in the presence of sodium nitroferricyanide as a catalyst^81^. The absorbance of indophenol blue at 660 nm was measured to determine the concentration of ammonia. N:P ratios were calculated as a single value averaged per site and season, owing to the different number of samples analyzed for ammonia.

Daily precipitation and river flow were obtained from the South Florida Water Management District’s DBHYDRO database and data from Station S-26, located on the Miami Canal (25.807628N, 80.260841W). Tidal height was obtained from the tidal station on Virginia Key (#8723214, NOAA/NOS/CO-OPS).

### Benthic community

Photomosaics were created at each site to capture and describe the benthic composition. Images were collected by divers swimming in a back and forth “lawnmower pattern” using two cameras (Hero 6 Black, GoPro) affixed to an aluminum bar (~ 60 cm apart) that were programmed to collect images every second. Field collection was conducted at MacArthur North in November 2018, Emerald Reef and MacArthur South in July 2019, and at Star Island in November 2019. Images were assembled into mosaics using Photoscan v1.3.4 (Agisoft). To determine the composition of benthic communities and substrate cover at each site, 98 0.25 m^2^ images were randomly sampled from each mosaic using ArcMap (VXX, ESRI). Fifty random points were overlaid on each image and the underlying taxon was manually identified using CoralNet^82^. To determine the species richness and size frequency of hard corals at each site, all mosaics were first cropped to a consistent 60 m^2^. Scleractinian corals were identified to species and their planar surface area was measured using spatial analysis tools in ArcMap.

### Fish composition

The community composition of fishes present in the Port of Miami was determined using photograph and video clips obtained from a live underwater camera (Octopus, View into the Blue) located off the Pilot House within the port (Fig. 1), and served at coralcitycamera.com. Fish monitoring was conducted from April 2020 to January 2021. These data are not directly tied to the benthic and environmental monitoring sites and simply describe the diverse communities of fishes present with the port. Fish abundances were categorized as abundant, seen on an hourly basis; common, seen on a daily basis; uncommon, seen on a weekly basis; rare, seen monthly or less; or seasonally common, observed as common on a seasonal basis.

### Data analysis

All data manipulation, calculations, and statistics were performed in R Studio^83^. Environmental and water quality data were analyzed using linear models with site and season as factors. Data were log transformed prior to analysis, with the exception of pH data which are already on a log scale. Time series of environmental data were produced using the *ggplot2* package^84^, and the frequencies of current vectors was plotted using the *openair* package^85^. Multivariate differences in benthic community composition across all benthic classes and with a filtered dataset of just scleractinian taxa were tested across sites using PERMANOVAs on square-root-transformed data in the *vegan* package^86^. SIMPERs were conducted in the same package to determine the benthic classes/taxa driving significant differences between sites, with a 70% cutoff threshold. Shannon diversity was calculated for datasets of all biota and for scleractinians only using relative abundance of benthic taxa (excluding abiotic classes), and compared among sites using one-way ANOVAs. Finally, size-frequency data were examined for the six most abundant scleractinian species across sites.

## Supplementary Information

Supplementary Information.

## Data Availability

Data produced as part of this study are publicly available at the National Centers for Environmental Information (NCEI, Accession 0276830) at https://www.ncei.noaa.gov/access/metadata/landing-page/bin/iso?id=gov.noaa.nodc:0276830.
