# Supplementary material for: Coral persistence despite marginal conditions in the Port of Miami
Source: Sci Rep. 2023 Apr 25;13:6759. doi: 10.1038/s41598-023-33467-7 (PMC10130011; doi:10.1038/s41598-023-33467-7)
Supplement: Supplementary file 1 — Supplementary Information. [file 41598_2023_33467_MOESM1_ESM.docx]

**Supplementary Figures and Tables**


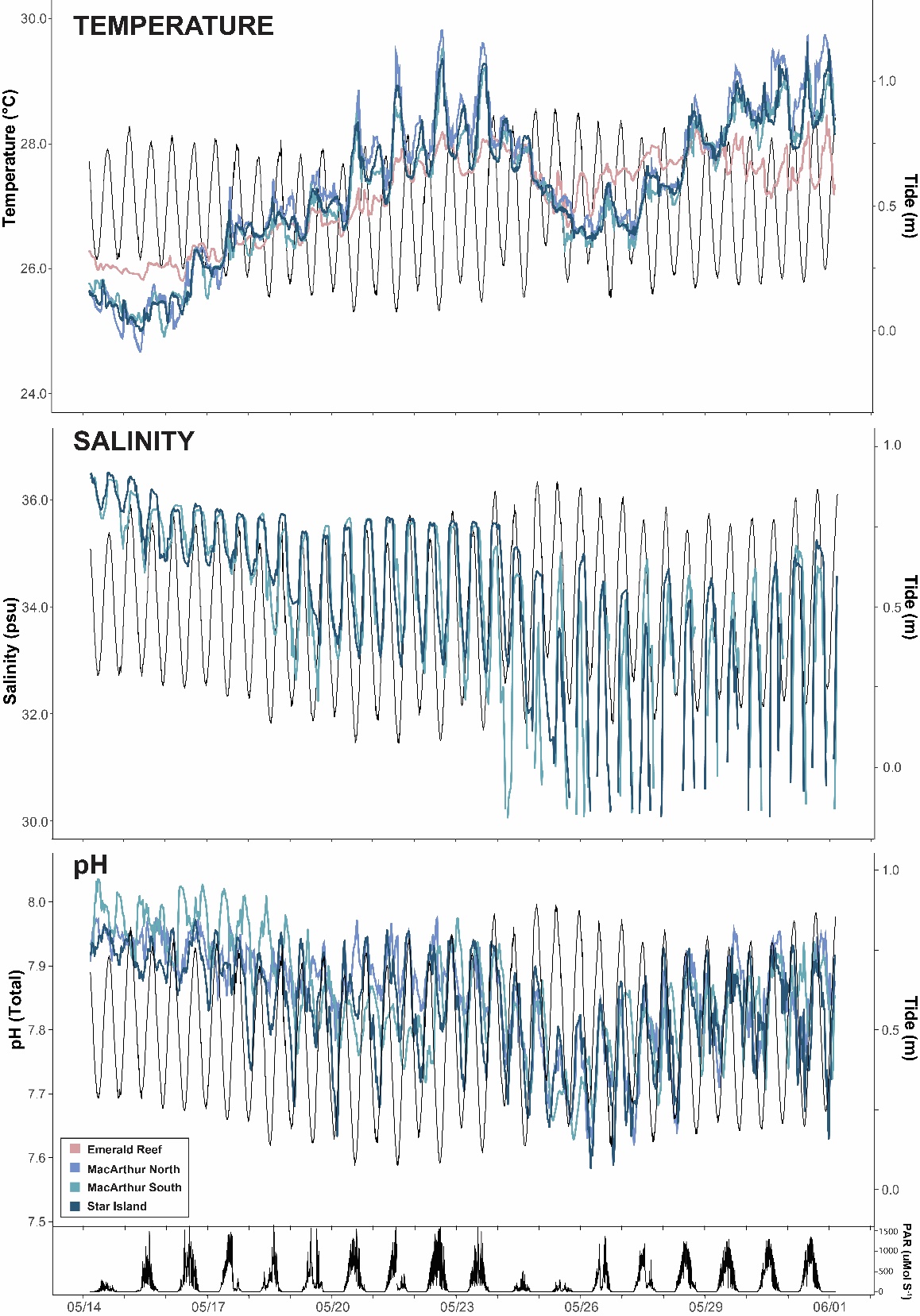


**Figure S1.** Temperature, salinity, and pH at a natural coral reef (pink) and three inshore urban sites (blue) over a period of two weeks. Tidal height (black) is overlaid on each plot. Instantaneous PAR (photosynthetically active radiation) is shown below.


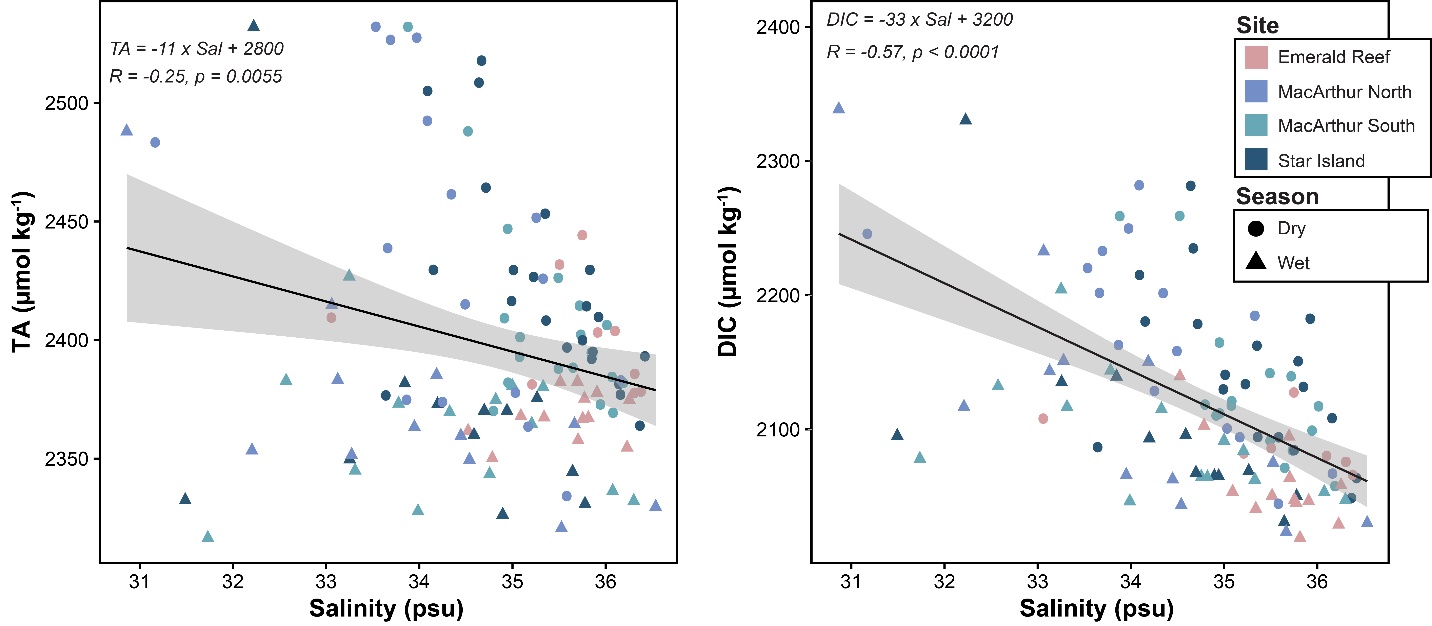


**Figure S2.** Total alkalinity (TA) and dissolved inorganic carbon (DIC) as a function of salinity. Colors denote sites with blue representing inshore urban environments and pink denoting natural Emerald Reef. Circles and triangles reflect samples collected in the dry and wet season, respectively. Linear regression is overlaid with the corresponding function, R, and p-value in the upper right of each plot.


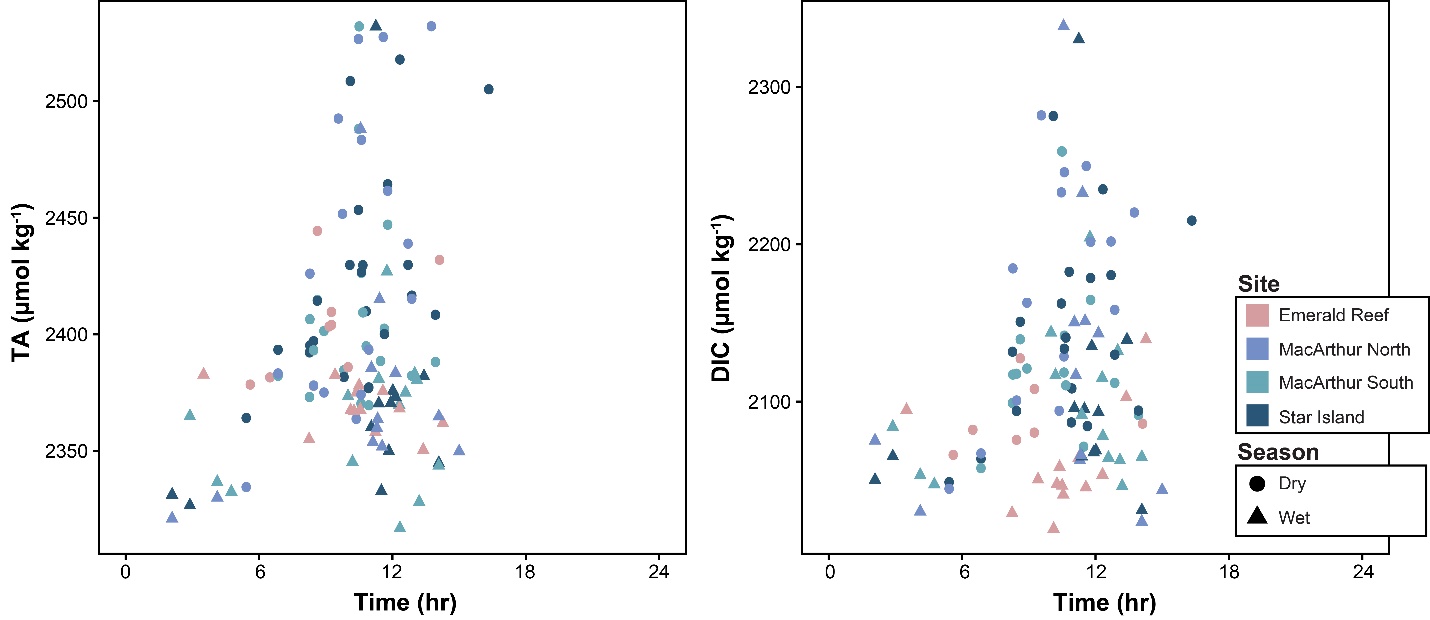


**Figure S3.** Total alkalinity (TA) and dissolved inorganic carbon (DIC) as a function of the time samples were collected. Colors denote sites with blue representing inshore urban environments and pink denoting natural Emerald Reef. Circles and triangles reflect samples collected in the dry and wet season, respectively.

**
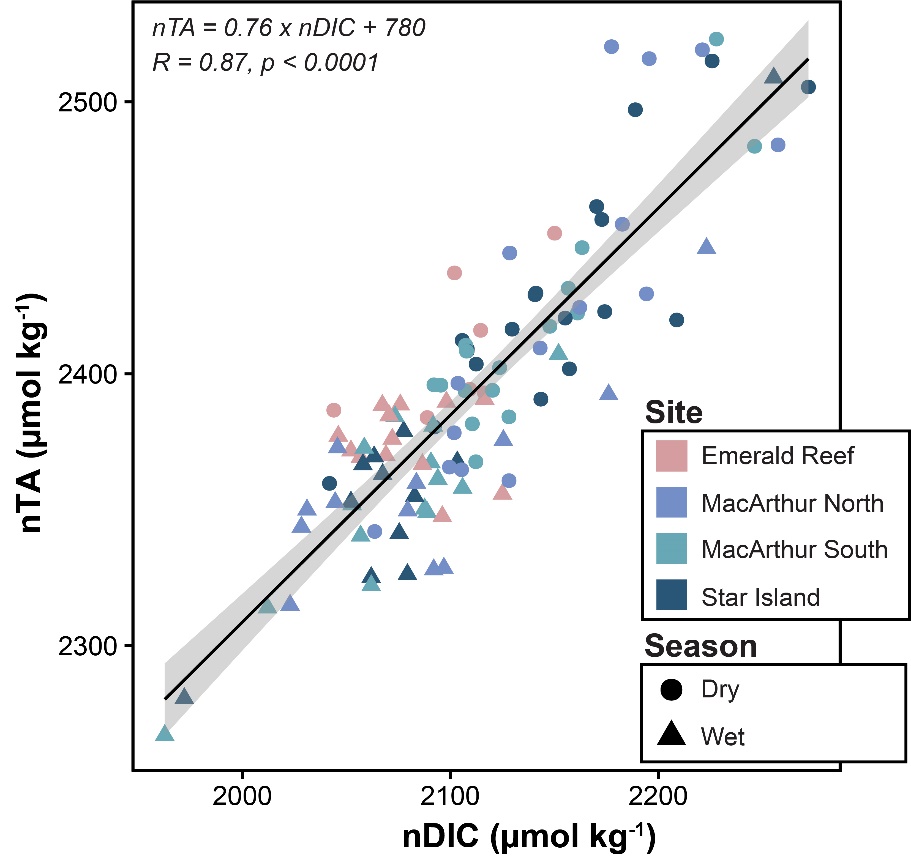
**

**Figure S4.** Property/property plot of salinity-normalized total alkalinity (nTA) and dissolved inorganic carbon (nDIC). Colors denote sites with blue representing inshore urban environments and pink denoting natural Emerald Reef. Circles and triangles reflect samples collected in the dry and wet season, respectively. Linear regression overlaid shown with the corresponding function, R, and p-value in the upper right of the plot.

**Table S1.** Linear model outputs for environmental parameters.

|  | **Df** | **SS** | **MS** | **F value** | **Pr(>F)** |
| --- | --- | --- | --- | --- | --- |
| **Temperature** | |  |  |  |  |
| Site | 3 | 11.6441 | 3.8814 | 779.1639 | <0.0001 |
| Season | 1 | 3565.2253 | 3565.2250 | 715698.8128 | <0.0001 |
| Residuals | 648287 | 3229.4160 | 0.0050 |  |  |
| **Salinity** |  |  |  |  |  |
| Site | 3 | 0.0234 | 0.0078 | 8.5616 | <0.0001 |
| Season | 1 | 0.0156 | 0.0156 | 17.0725 | 0.0001 |
| Residuals | 131 | 0.1193 | 0.0009 |  |  |
| **PAR** |  |  |  |  |  |
| Site | 3 | 60.6540 | 20.2180 | 23.1603 | <0.0001 |
| Season | 1 | 7.5853 | 7.5853 | 8.6892 | 0.0033 |
| Residuals | 967 | 844.1513 | 0.8730 |  |  |
| **Flow** |  |  |  |  |  |
| Site | 3 | 46110.3737 | 15370.1200 | 25425.3088 | <0.0001 |
| Season | 1 | 36.9149 | 36.9149 | 61.0647 | <0.0001 |
| Residuals | 100926 | 61011.8525 | 0.6045 |  |  |

**Table S2.** Linear model outputs for carbonate chemistry data. The pH and pH Spec models are run on instrument-data and discrete bottle samples analyzed using a spectrophotometer, respectively. Models are run on both total alkalinity (TA) and dissolved inorganic carbon (DIC), as well as on values that have been salinity-normalized, denoted as nTA and nDIC respectively. Data for the models run on the partial pressure of CO_2_ *(p*CO_2_) and the saturation state of aragonite (Ω_Arag_) are calculated from TA and DIC.

|  | **Df** | **SS** | **MS** | **F value** | **Pr(>F)** |
| --- | --- | --- | --- | --- | --- |
| **pH** |  |  |  |  |  |
| Site | 3 | 75.1577 | 25.0526 | 4140.9780 | 0.0000 |
| Season | 1 | 152.5599 | 152.5599 | 25216.8730 | 0.0000 |
| Residuals | 52075 | 315.0492 | 0.0060 |  |  |
| **pH Spec** |  |  |  |  |  |
| Site | 3 | 0.0576 | 0.0192 | 6.0811 | 0.0007 |
| Season | 1 | 0.1861 | 0.1861 | 58.8955 | <0.0001 |
| Residuals | 119 | 0.3760 | 0.0032 |  |  |
| **TA** |  |  |  |  |  |
| Site | 3 | 0.0021 | 0.0007 | 2.2339 | 0.0881 |
| Season | 1 | 0.0118 | 0.0118 | 38.0147 | <0.0001 |
| Residuals | 113 | 0.0351 | 0.0003 |  |  |
| **nTA** |  |  |  |  |  |
| Site | 3 | 0.0010 | 0.0003 | 1.1907 | 0.3166 |
| Season | 1 | 0.0169 | 0.0169 | 60.8260 | <0.0001 |
| Residuals | 113 | 0.0314 | 0.0003 |  |  |
| **DIC** |  |  |  |  |  |
| Site | 3 | 0.0158 | 0.0053 | 6.4688 | 0.0005 |
| Season | 1 | 0.0092 | 0.0092 | 11.2912 | 0.0011 |
| Residuals | 103 | 0.0840 | 0.0008 |  |  |
| **nDIC** | |  |  |  |  |
| Site | 3 | 0.0041 | 0.0014 | 2.5275 | 0.0615 |
| Season | 1 | 0.0210 | 0.0210 | 38.7000 | <0.0001 |
| Residuals | 103 | 0.0558 | 0.0005 |  |  |
| **pCO_2_** |  |  |  |  |  |
| Site | 3 | 0.4216 | 0.1405 | 5.3071 | 0.0019 |
| Season | 1 | 1.0176 | 1.0176 | 38.4235 | <0.0001 |
| Residuals | 103 | 2.7278 | 0.0265 |  |  |
| **Omega** |  |  |  |  |  |
| Site | 3 | 0.2418 | 0.0806 | 7.1078 | 0.0002 |
| Season | 1 | 0.0043 | 0.0043 | 0.3784 | 0.5398 |
| Residuals | 103 | 1.1679 | 0.0113 |  |  |

**Table S3.** Linear model outputs for nutrient data.

|  | **Df** | **SS** | **MS** | **F value** | **Pr(>F)** |
| --- | --- | --- | --- | --- | --- |
| **Si** |  |  |  |  |  |
| Site | 3 | 7.7688 | 2.5896 | 10.0776 | <0.0001 |
| Season | 1 | 1.3685 | 1.3685 | 5.3256 | 0.0247 |
| Residuals | 57 | 14.6470 | 0.2570 |  |  |
| **NO_2_** |  |  |  |  |  |
| Site | 3 | 0.0336 | 0.0112 | 2.8092 | 0.0475 |
| Season | 1 | 0.0223 | 0.0223 | 5.5971 | 0.0214 |
| Residuals | 57 | 0.2270 | 0.0040 |  |  |
| **NO_3_** |  |  |  |  |  |
| Site | 3 | 0.5664 | 0.1888 | 4.3107 | 0.0083 |
| Season | 1 | 0.0445 | 0.0445 | 1.0161 | 0.3177 |
| Residuals | 57 | 2.4966 | 0.0438 |  |  |
| **PO_4_** |  |  |  |  |  |
| Site | 3 | 0.0625 | 0.0208 | 0.6860 | 0.5643 |
| Season | 1 | 0.0859 | 0.0859 | 2.8303 | 0.0980 |
| Residuals | 57 | 1.7301 | 0.0304 |  |  |
| **NH_4_** |  |  |  |  |  |
| Site | 3 | 0.2496 | 0.0832 | 0.1147 | 0.9510 |
| Season | 1 | 6.2615 | 6.2615 | 8.6318 | 0.0053 |
| Residuals | 42 | 30.4667 | 0.7254 |  |  |

**Table S4.** Linear model outputs for benthic cover data.

|  | **Df** | **SS** | **MS** | **F value** | **Pr(>F)** |
| --- | --- | --- | --- | --- | --- |
| **Abiotic** |  |  |  |  |  |
| Site | 3 | 6.1557 | 2.0519 | 18.3471 | <0.0001 |
| Residuals | 388 | 43.3927 | 0.1118 |  |  |
| **Macroalgae** |  |  |  |  |  |
| Site | 3 | 0.4684 | 0.1561 | 37.9387 | <0.0001 |
| Residuals | 388 | 1.5967 | 0.0041 |  |  |
| **Turf algae** |  |  |  |  |  |
| Site | 3 | 3.0517 | 1.0172 | 9.4718 | <0.0001 |
| Residuals | 388 | 41.6690 | 0.1074 |  |  |
| **CCA** |  |  |  |  |  |
| Site | 3 | 0.0000 | 0.0000 | 2.0206 | 0.1105 |
| Residuals | 388 | 0.0009 | 0.0000 |  |  |
| **Clionaid sponges** | |  |  |  |  |
| Site | 3 | 0.1238 | 0.0413 | 16.9913 | <0.0001 |
| Residuals | 388 | 0.9420 | 0.0024 |  |  |
| **Hard coral** |  |  |  |  |  |
| Site | 3 | 0.2431 | 0.0810 | 9.8435 | <0.0001 |
| Residuals | 388 | 3.1940 | 0.0082 |  |  |
| **Cyanobacteria** |  |  |  |  |  |
| Site | 3 | 0.0003 | 0.0001 | 4.4399 | 0.0044 |
| Residuals | 388 | 0.0087 | 0.0000 |  |  |
| **Fire coral** |  |  |  |  |  |
| Site | 3 | 0.0000 | 0.0000 | 2.0199 | 0.1106 |
| Residuals | 388 | 0.0009 | 0.0000 |  |  |
| **Soft coral** |  |  |  |  |  |
| Site | 3 | 1.5187 | 0.5062 | 39.3941 | <0.0001 |
| Residuals | 388 | 4.9858 | 0.0129 |  |  |
| **Sponge** |  |  |  |  |  |
| Site | 3 | 0.0720 | 0.0240 | 12.0984 | <0.0001 |
| Residuals | 388 | 0.7696 | 0.0020 |  |  |
| **Other** |  |  |  |  |  |
| Site | 3 | 0.0884 | 0.0295 | 7.6618 | <0.0001 |
| Residuals | 388 | 1.4927 | 0.0038 |  |  |

**Table S5.** Fishes observed at the Coral City Camera site organized by family. Frequency of occurrence as follows: A, Abundant (seen on a hourly basis); C, Common (seen on an daily basis); O, Occasional (seen on a weekly basis); U, Uncommon (seen on a monthly basis); R, Rare (1-5 sightings); SC, Seasonally Common, rare at other times.

| **Type of Fish** | **Scientific Name** | **Common Name** | **Frequency** |
| --- | --- | --- | --- |
| **Acanthuridae** | *Acanthurus bahianus* | Barber surgeonfish | C |
|  | *Acanthurus chirugus* | Doctorfish | A |
|  | *Acanthurus coeruleus* | Blue tang | O |
| **Achiridae** | *Trinectes maculatus* | Hogchoker | R |
| **Aetobatidae** | *Aetobatus narinari* | Spotted eagle ray | U |
| **Balistidae** | *Balistes capriscus* | Gray triggerfish | R |
|  | *Canthidermis sufflamen* | Ocean triggerfish | R |
| **Belonidae** | *Tylosurus crocodilus* | Houndfish | C |
| **Bothidae** | *Bothus ocellatus* | Eyed flounder | R |
|  | *Engyophrys senta* | Spiny flounder | R |
| **Carangidae** | *Caranx bartholomaei* | Yellow jack | C |
|  | *Caranx crysos* | Blue runner | O |
|  | *Caranx hippos* | Crevalle jack | C |
|  | *Caranx latus* | Horse-eye jack | O |
|  | *Caranx ruber* | Bar jack | A |
|  | *Decapterus punctatus* | Round scad | SC |
|  | *Selene vomer* | Lookdown | R |
|  | *Trachinotus falcatus* | Permit | R |
|  | *Uraspis secunda* | Cottonmouth jack | R |
| **Carcharhinidae** | *Carcharhinus acronotus* | Blacknose shark | R |
|  | *Carcharhinus limbatus* | Blacktip shark | U (SC) |
|  | *Negaprion brevirostris* | Lemon shark | U (SC) |
| **Centropomidae** | *Centropomus undecimalis* | Common snook | U |
| **Chaetodontidae** | *Chaetodon capistratus* | Foureye butterflyfish | A |
|  | *Chaetodon ocellatus* | Spotfin butterflyfish | C |
| **Clupeidae** | *Harengula jaguana* | Scaled herring | SC |
|  | *Sardinella aurita* | Round sardinella | SC |
| **Cyclopsettidae** | *Syacium micrurum* | Channel flounder | U |
| **Dasyatidae** | *Hypanus americanus* | Southern stingray | O |
|  | *Hypanus say* | Bluntnose ray | U |
| **Diodontidae** | *Diodon holocanthus* | Balloonfish | U |
|  | *Diodon hysterix* | Porcupinefish | O |
| **Echeneidae** | *Echeneis naucrates* | Live sharksucker | U |
| **Ephippidae** | *Chaetodipterus faber* | Atlantic spadefish | C |
| **Fistulariidae** | *Fistularia tabacaria* | Cornetfish | R |
| **Gerreidae** | *Eucinostomus argenteus* | Spotfin mojarra | O |
|  | *Eucinostomus havana* | Bigeye mojarra | O |
|  | *Eucinostomus lefroyi* | Mottled mojarra | O |
|  | *Eucinostomus melanopterus* | Flagfin mojarra | O |
|  | *Gerres cinereus* | Yellowfin mojarra | C |
| **Ginglymostomatidae** | *Ginglyostoma cirratum* | Nurse shark | O |
| **Gobiidae** | *Bathygobius soporator* | Frillfin goby | U |
|  | *Coryphopterus glaucofraenum* | Bridled goby | C |
|  | *Gnatholepis thompsoni* | Goldspot goby | C |
| **Haemulidae** | *Anisotremus surinamensis* | Black margate | U |
|  | *Anisotremus virginicus* | Porkfish | C |
|  | *Haemulon aurolineatum* | Tomtate | A |
|  | *Haemulon carbonium* | Caesar grunt | R |
|  | *Haemulon chrysargyreum* | Smallmouth grunt | C |
|  | *Haemulon flavolineatum* | French grunt | A |
|  | *Haemulon macrostomum* | Spanish grunt | U |
|  | *Haemulon parra* | Sailor's grunt | U |
|  | *Haemulon plumierii* | White grunt | A |
|  | *Haemulon sciurus* | Bluestriped grunt | C |
| **Kyphosidae** | *Kyphosus sectatrix* | Bermuda chub | A |
|  | *Kyphosus vaigiensis* | Yellow chub | C |
| **Labridae** | *Bodianus rufus* | Spanish hogfish | U |
|  | *Halichoeres bivittatus* | Slippery dick | A |
|  | *Halichoeres maculipinna* | Clown wrasse | O |
|  | *Halichoeres poeyi* | Blackear wrasse | R |
|  | *Halichoeres radiatus* | Puddingwife | O |
|  | *Lachnolaimus maximus* | Hogfish | C |
|  | *Thalassoma bifasciatum* | Bluehead wrasse | O |
| **Labrisomidae** | *Malacoctenus macropus* | Rosy blenny | O |
| **Lutjanidae** | *Lutjanus analis* | Mutton snapper | O |
|  | *Lutjanus apodus* | Schoolmaster | C |
|  | *Lutjanus cyanopterus* | Cubera snapper | O |
|  | *Lutjanus griseus* | Gray snapper | A |
|  | *Lutjanus jocu* | Dog snapper | O |
|  | *Lutjanus mahogoni* | Mahogany snapper | U |
|  | *Lutjanus synagris* | Lane snapper | U |
|  | *Ocyurus chrysurus* | Yellowtail snapper | C |
| **Megalopidae** | *Megalops atlanticus* | Tarpon | U |
| **Mobulidae** | *Mobula hypostoma* | Devil ray | R |
| **Monacanthidae** | *Aluterus schoepfii* | Orange filefish | R |
|  | *Aluterus scriptus* | Scawled filefish | C |
|  | *Cantherhines pullus* | Orangespotted filefish | C |
|  | *Monacanthus tuckeri* | Slender filefish | R |
|  | *Stephanolepis hispidus* | Planehead filefish | R |
|  | *Stephanolepis setifer* | Pygmy filefish | O |
| **Muglidae** | *Mugil curema* | White mullet | SC |
| **Mullidae** | *Mulloidichthys martinicus* | Yellow goatfish | O |
|  | *Pseudupeneus maculatus* | Spotted goatfish | O |
| **Muraenidae** | *Gymnothorax funebris* | Green moray | U |
|  | *Gymnothorax vicinus* | Purplemouth moray | R |
| **Ophichthidae** | *Myrichthys breviceps* | Sharptail eel | R |
| **Ostraciidae** | *Acanthostracion quadricornis* | Scrawled cowfish | U |
|  | *Lactophrys bicaudalis* | Spotted trunkfish | O |
|  | *Lactophrys trigonus* | Buffalo trunkfish | U |
|  | *Lactophrys triqueter* | Smooth trunkfish | O |
| **Pomacanthidae** | *Holacanthus bermudensis* | Blue angelfish | R |
|  | *Holocanthus ciliaris* | Queen angelfish | U |
|  | *Pomacanthus arcuatus* | Gray angelfish | A |
|  | *Pomacanthus paru* | French angelfish | C |
| **Pomacentridae** | *Abudefduf saxatilis* | Sergeant major | A |
|  | *Abudefduf taurus* | Night sergeant | O |
|  | *Microspathodon chrysurus* | Yellowtail damselfish | O |
|  | *Stegastes adustus* | Dusky damselfish | A |
|  | *Stegastes partitus* | Bicolor damselfish | C |
|  | *Stegastes variabilis* | Cocoa damselfish | C |
| **Pristidae** | *Pristis pectinata* | Smalltooth sawfish | R |
| **Rhinobatidae** | *Rhinobatos lentiginosus* | Atlantic guitarfish | R |
| **Scaridae** | *Scarus coelestinus* | Midnight parrotfish | C |
|  | *Scarus coeruleus* | Blue parrotfish | C |
|  | *Scarus guacamaia* | Rainbow parrotfish | C |
|  | *Scarus iserti* | Striped parrotfish | A |
|  | *Scarus vetula* | Queen parrotfish | O |
|  | *Sparisoma atomarium* | Greenblotch parrotfish | R |
|  | *Sparisoma aurofrenatum* | Redband parrotfish | A |
|  | *Sparisoma chrysopterum* | Redtail parrotfish | A |
|  | *Sparisoma rubripinne* | Yellowtail parrotfish | A |
|  | *Sparisoma viride* | Stoplight parrotfish | A |
| **Sciaenidae** | *Pareques acuminatus* | Highhat | R |
| **Scombridae** | *Scomberomorus regalis* | Cero | R |
| **Scorpaenidae** | *Pterois* sp. | Lionfish | U |
|  | *Scorpaena plumieri* | Spotted scorpionfish | O |
| **Serranidae** | *Cephalopholis cruentatus* | Graysby | R |
|  | *Diplectrum formosum* | Sand perch | R |
|  | *Hypoplectrus puella* | Barred hamlet | O |
|  | *Hypoplectrus unicolor* | Butter hamlet | R |
|  | *Mycteroperca bonaci* | Black grouper | U |
| **Sparidae** | *Archosargus probatocephalus* | Sheepshead | R |
|  | *Archosargus rhomboidalis* | Sea bream | U |
|  | *Calamus calamus* | Saucereye porgy | U |
|  | *Calamus leucosteus* | Whitebone porgy | U |
|  | *Calamus penna* | Sheepshead porgy | O |
|  | *Calamus pennatula* | Pluma | O |
|  | *Diplodus argenteus* | Silver porgy | O |
|  | *Diplodus holbrookii* | Spottail porgy | C |
|  | *Lagodon rhomboides* | Pinfish | U |
| **Sphyraenidae** | *Sphyraena barracuda* | Great barracuda | C |
| **Sphyrnidae** | *Sphyrna tiburo* | Bonnethead shark | R |
| **Synodontidae** | *Synodus foetens* | Inshore lizardfish | R |
|  | *Synodus intermedius* | Sand diver | O |
| **Tetraodontidae** | *Canthigaster rostrata* | Sharpnose puffer | A |
|  | *Sphoeroides nephalus* | Southern puffer | R |
|  | *Sphoeroides spengleri* | Bandtail puffer | C |
|  | *Sphoeroides testudineus* | Checkered puffer | U (SC) |
| **Urotrygonidae** | *Urolophus jamaicensis* | Yellow stingray | C |
